# Use of Colomba-Root against Vomiting

**Published:** 1844-11

**Authors:** 


*Use of Colomba-Root against Vomiting.*—This well known and long established drug is an admirable remedy in many cases of ataxic and nervous vomiting. A writer in a recent number of the Annali Universali, mentions the case of a soldier, who was suddenly seized, after a fright, with violent pain in the abdomen, accompanied with vomiting and diarrhœa. The other symptoms gradually ceased; but the vomiting, (which was evidently of a nervous character,) still continued. The patient had a yellow cachectic look, a furred white tongue, and loss of appetite; slight epigastric pain usually preceded the coming on of the fits of vomiting. They generally returned six or seven times every day. Every thing tried failed in checking this obstinate irritability of the stomach, until seven months after the commencement of the disease—Dr. Monfredonia suggested the use of Colomba; it was given in the form of powder, and in the dose of eight grains four times in the 24 hours, in a small cupful of goats' milk. A very sensible amendment in the state of the patient speedily took place, but the vomiting did not entirely cease for some time. The Colomba was subsequently given in the form of the decoction; and under its use the health was gradually, but completely restored.

Colomba is an excellent remedy in a great many cases of gastric ailment: a favorite formula of ours is—Infusi Colombæ ℥ vj., Liq. Potassæ ʒ iss., Tinct. Humuli ʒ iij., Aquæ Pimentæ ℥ iss. M. It is often very useful in phthisical patients.—*Ibid.*

